# β-tricalcium phosphate/gelatin composite scaffolds incorporated with gentamycin-loaded chitosan microspheres for infected bone defect treatment

**DOI:** 10.1371/journal.pone.0277522

**Published:** 2022-12-08

**Authors:** Yu Liu, Qingqing Zhao, Changsong Chen, Chunhu Wu, Yuhai Ma

**Affiliations:** 1 Department of Orthopedics, Hospital of Zhejiang People’s Armed Police (PAP), Hangzhou, Zhejiang, China; 2 Zhejiang Zhongwei Medical Research Center, Hangzhou, Zhejiang, China; College of Engineering, University of Saskatchewan, CANADA

## Abstract

The repair of infected bone defects remains a clinical challenge. *Staphylococcus aureus* is a common pathogenic micro-organism associated with such infections. Gentamycin (GM) is a broad spectrum antibiotic that can kill *S*. *aureus* in a dose-dependent manner. However, the systemic administration of antibiotics may lead to drug resistance and gut dysbiosis. In this work, we constructed β-tricalcium phosphate/gelatin composite scaffolds incorporated with gentamycin-loaded chitosan microspheres (CMs(GM)-β-TCP/gelatin composite scaffolds), which helped optimize the local GM release in the infected defect areas and enhance bone regeneration. The cumulative release curves showed that both microspheres and composite scaffolds reached a sustained slow-release phase after the initial rapid release, and the latter further stabilized the initial drug release rate. The release curve of CMs(GM)-β-TCP/gelatin composite scaffolds reached a plateau after 24 h, and the cumulative release reached 41.86% during this period. Moreover, the combination of β-TCP and gelatin mimicked bone composition and were able to provide the requisite mechanical strength (0.82 ± 0.05 MPa) during the first phase of bone generation. The inner structure of the scaffold was arranged in the shape of interconnected pores, and presented a porosity level of 16%. The apertures were uniform in size, which was beneficial for cell proliferation and material transportation. Macroscopic observation and histological analysis showed that CMs(GM)-β-TCP/gelatin composite scaffolds fused with bone tissues, and new tissues were formed in defect areas without any infection. This new composite scaffold may be a promising repair material for treating infected bone defects.

## Introduction

Bone infections following open bone fractures increase the difficulty of defect treatment and functional recovery [1]. Invading bacteria and other pathogens can colonize the bone and cause the release of osteolytic cytokines, which subsequently leads to bone destruction [2]. Moreover, the production of immune mediators related to infection may affect the function of osteoblasts and osteoclasts [2,3]. Currently, systemic administration of antibiotics, both orally and intravenously, is an effective treatment for controlling microbial infection [4]. Gentamycin (GM) is a broad spectrum aminoglycoside antibiotic that exhibits a strong inhibitory effect on gram-positive and gram-negative bacteria [5–7]. Moreover, GM can kill *Staphylococcus aureus* infecting human osteosarcoma cells SAOS-2 in a dose-dependent manner [6]. *S*. *aureus* is a common pathogenic micro-organism associated with bone infections [8,9]. However, the systemic administration of antibiotics has limitations such as poor permeability and low concentrations of drugs in target tissues [10]. Moreover, large doses of antibiotics can cause serious adverse effects [10]. For example, gut microbial regrouping following the administration of oral broad spectrum antibiotics can lead to gut dysbiosis and induce bone loss [11].

The use of biological materials as drug carriers is the main method to reduce toxic side effects and improve the therapeutic effect [12,13]. Microspheres (MSs) are microscale tailorable platforms that can enhance the biological distribution and site-specific targeted delivery of drugs [13]. Chitosan (CS) is a common candidate for MS fabrication. CS is a natural polysaccharide that is biocompatible and economic [14,15]. More importantly, CS has pharmacological effects such as bactericidal and antibacterial activities [14,16]. Two mechanisms have been proposed to explain the antibacterial activity of CS: (i) Electrostatic attraction between the positive charges carried by the CS chain and the negative charges on the bacterial cell wall, resulting in the destruction of the stable structure of the cell membrane and leakage of intracellular components; (ii) enclosing of bacteria by the polymer formed by CS, preventing the exchange and absorption of materials [16,17].

Small MSs may be easily cleared by capillaries and lymphatic vessels. Only when the number of MSs is sufficiently large can a sufficient therapeutic concentration of the drug be maintained [18]. However, a large dose can increase the probability of adverse effects [18]. For bone fractures that cannot heal completely over their natural lifetime, the mere application of MSs is also insufficient [19,20]. Standard treatment protocol of bone infection requires grafting materials as bone void fillers after timely surgical debridement and antibiotic therapy. Polymethylmethacrylate (PMMA) has become the most commonly used filler of bone defect, although it is not biodegradable and subsequent surgeries are often needed [21]. In recent years, a variety of biodegradable materials have been developed to fill osseous defects. Boyle et al. developed a calcium phosphate-calcium sulfate composite promoting bone formation [22]. Beta-tricalcium phosphate (β-TCP) is a good candidate because it is an important inorganic component of natural bone tissue with excellent biological activity and osteoconductive and osteoinductive properties [19]. Beta-TCP can be degraded *in vivo* and provides appropriate space for bone restoration [23]. However, β-TCP has inherent shortcomings such as poor toughness, high brittleness, and high elastic modulus when used alone. Gelatin is a natural mixture containing peptides and proteins derived from the hydrolysis of collagen and has the advantages of being economic, easy production, and low immunogenicity [24]. Gelatin retains the Arg-Gly-Asp (RGD) peptide to provide a molecular binding domain for interactions with subsets of host cells, which promotes cell adhesion and bone reconstruction [25–27]. It can be hydrolyzed by enzymes *in vivo*, and its degradation rate is higher than expected [25].

**Fig 1:**
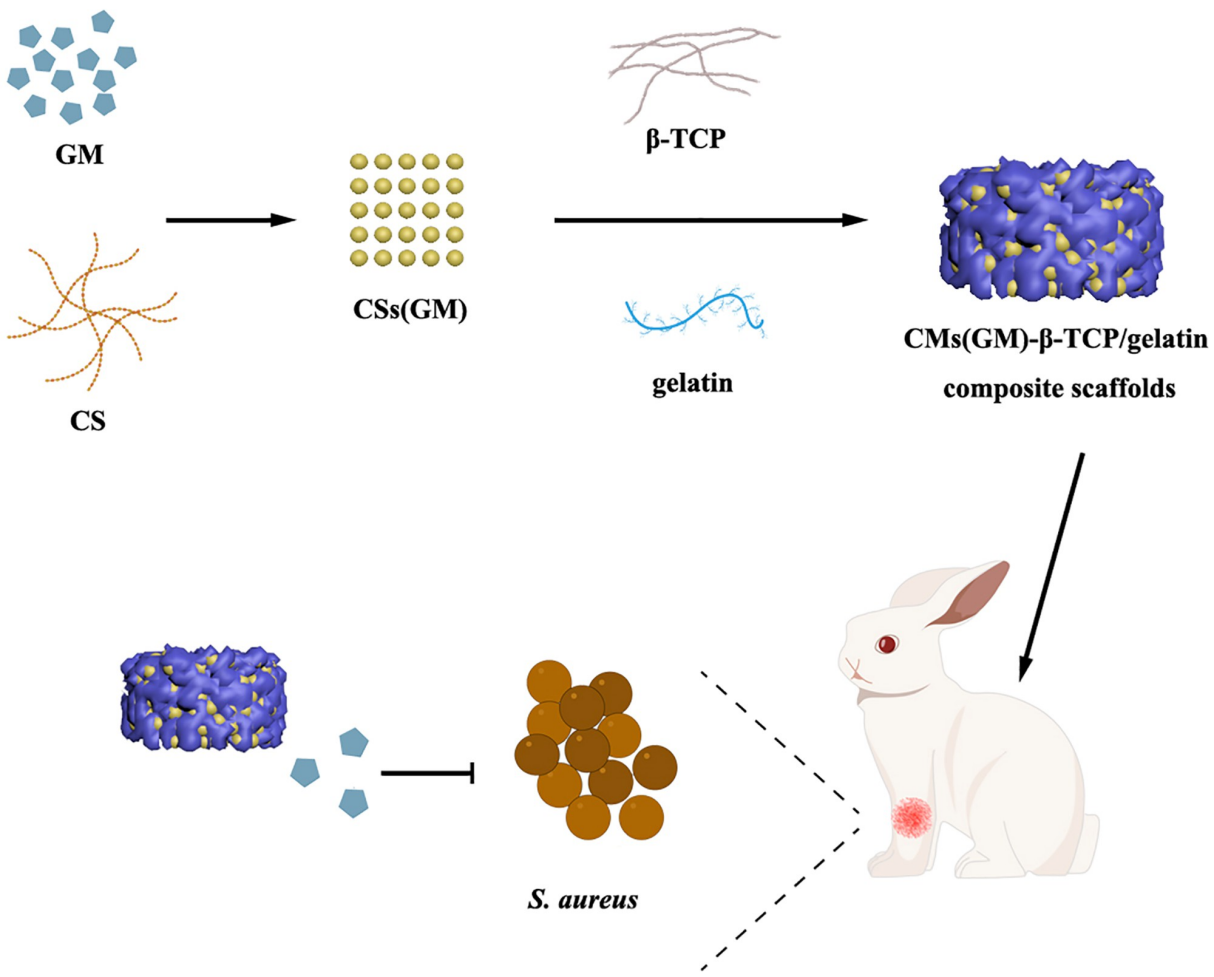
Preparation and mechanism of CMs(GM)-β-TCP/gelatin composite scaffolds (CMs: Chitosan microspheres; GM: Gentamycin; β-TCP: β-tricalcium phosphate; CS: Chitosan; *S*. *aureus*: *Staphylococcus aureus*).

In this study, we fabricated β-TCP/gelatin composite scaffolds incorporated with CMs(GM) (CMs(GM)-β-TCP/gelatin composite scaffolds) to optimize local delivery of GM and provide a biodegradable void filler of bone in the treatment of bone infections (see Fig 1). The design of this composite scaffold realizes the complementary advantages of β-TCP and gelatin, which share similarities with human bone tissue. This study was performed to characterize the morphology and structure of CS(GM)/β-TCP/gelatin composite scaffolds to support GM release *in vitro* and to evaluate the effects of implantation of CMs(GM)-β-TCP/gelatin composite scaffolds on radial segmental bone defects accompanied by infection *in vivo*.

## Materials and methods

### Materials and reagents

CS was purchased from Shanghai Yuanye Biotechnology Co., Ltd. (Shanghai, China). GM, genipin, β-TCP, and boric acid were purchased from Shanghai Macklin Biochemical Co., Ltd. (Shanghai, China). Gelatin was purchased from Beijing Solarbio Science and Technology Co., Ltd. (Beijing, China). Hematoxylin and eosin were purchased from Sigma Aldrich Trading Co., Ltd. (Shanghai, China). Methyl methacrylate was purchased from Shanghai Zhanyun Chemical Co. Ltd. (Shanghai, China). Paraformaldehyde was purchased from Shanghai Lingfeng Chemical Reagent Co. Ltd. (Shanghai, China). Acetic and hydrochloric acids were purchased from Xilong Scientific Co., Ltd. (Guangdong, China).

### Animals

Fifteen female New Zealand white rabbits (4–5-month-old, 2.5–3.0 kg) were randomly divided into control group (n = 6), infected group (n = 6), and experimental group (n = 3). Three rabbits in the control group and three in the infected group were sacrificed to determine whether the infection model was constructed successfully. The rabbits were acclimatized for one week before the experiment. The rabbits were housed under standard laboratory conditions with a 12-h/12-h light/dark cycle and provided ad libitum access to water and food. The temperature and relative humidity were controlled at 20–25°C and 40–60%, respectively. The study protocol was approved by the Laboratory Animal Welfare and Ethics Committee of Institute of Basic Medicine and Cancer, Chinese Academy of Science.

### Fabrication of CMs(GM)

CMs(GM) were fabricated using the spray drying method after stirring and dissolving 10 g of CS and 5 g of GM in 500 mL of 1% acetic acid solution. Blank MSs were prepared using the same procedure.

### Fabrication of CMs(GM)-β-TCP/gelatin composite scaffolds

To prepare the suspensions, 1.8 g of CMs(GM) and 1.1 g of β-TCP were added to 5 mL of 18% gelatin solution at 37°C. After constant stirring at 5000 rpm for 5 min, 1.5 mL of 1% genipin solution was added and stirred for another 30 min. The mixture was poured into cylindrical molds (diameter: 4 mm; length: 10 mm) and freeze-dried overnight. The obtained scaffolds were trimmed into a cylinder (4 mm diameter; 10 mm height) and sterilized with ethylene oxide. The composite scaffolds were stored at −20°C for later experiments.

### Determination of encapsulation efficiency and drug loading of microspheres

The concentration of GM in CMs(GM) was determined using derivative ultraviolet spectrophotometry. First, several concentrations of GM standard solutions were prepared. Boric acid–acetic acid and acetyl acetone–formaldehyde were used as the buffer and indicator, respectively. A standard curve was plotted with the concentration along the x-axis and the absorbance at 339 nm along the y-axis. Subsequently, 0.5 g of CMs(GM) were fully ground and dissolved in 2 mL phosphoric acid buffer (pH = 8.0). After 24 h of standing and derivatization, the GM content in the samples (W_1_) was calculated using the standard curve formula. The encapsulation efficiency and drug loading of GM in the CS MSs were calculated using the following formulas:

$$\text{Encapsulation}\;\text{efficiency}\;\left(\text{EF\%}\right)\;=\;{\text{W}}_{\text{1}}\text{/}{\text{W}}_{\text{2}}\times 100\%$$

and

$$\text{Drug}\;\text{loading}\;\left(\text{DL\%}\right)\;=\;{\text{W}}_{\text{1}}\text{/}{\text{W}}_{\text{3}}\times 100\%$$

where W_2_ and W_3_ represent the total volumes of GM and CMs(GM), respectively.

### Evaluation of bactericidal activity of microspheres

The agar diffusion method was used to evaluated the antimicrobial property of the CMs(GM). As model bacteria, *S*. *aureus* (1 × 10^8^ CFU mL^-1^) was seeded over agar plate. Eight 6-mm filter paper disks were evenly spaced upon the agar, five of which contained 15, 20, 25, 30, and 35 μg mL^-1^ GM, respectively. The remaining three papers contained the leachate of CMs(GM) at different dilutions. Briefly, 100 mg CMs(GM) were placed in dialysis bags, and 100 mL of phosphoric acid buffer (pH = 8.0) was added. CMs(GM) were leached for 24 h on a shaker. The leachate was further diluted 2-fold and 4-fold. Subsequently, the inhibitory halo was observed to evaluate the bactericidal activity of microspheres.

### Scanning electron microscopy analysis of microspheres and scaffolds

The morphology and structure of the MSs and composite scaffolds were characterized using scanning electron microscopy (SEM). Briefly, the composite scaffold specimens were sliced into thin sections and fixed onto the platform. The MSs were then fixed directly onto the platform. After sputter-coating with gold, the sections were examined using a Hitachi SEM (TM-1000).

### Evaluation of the mechanical properties of the scaffolds

In order to study the mechanical behavior of the scaffolds, uniaxial compression assays were performed with a crosshead speed of 1 mm min^-1^, using a Universal Material Testing Machine (Jiangsu Hengguang Precision Instrument Co., Ltd, China). The samples were cylindrical molds of 4 mm in diameter and 10 mm in length. Three samples of each scaffold were tested and compressive strength (Cs) was determined using the following formulas:

$$\text{Compressive}\;\text{strength}\;(\text{Cs})\;=\;\frac{\text{F}}{\text{A}}$$

where F is the load at the time of the fracture and A represents the contact area of the scaffold.

### Porosity evaluation of composite scaffolds

The total porosity (P) of the composite scaffolds was determined using the liquid displacement method. First, the initial dry weight (W_1_) of the dry scaffold was measured. Then the scaffold was placed in absolute ethanol (EtOH) for 48 h. EtOH was used as the displacement liquid due to its ability to permeate the pores easily without swelling or shrinking. Subsequently, the final weight of the wet scaffold was measured and the porosity was calculated using the following formulas:

$$\text{P(\%)}\;=\;\frac{{\text{W}}_{\text{2}}-{\text{W}}_{\text{1}}}{\rho \times \text{V}}$$

where ρ represent the density of the EtOH, and V represent the volume of the scaffold, which is directly determined by immersion.

### Fourier transform infrared spectroscopy analysis

To examine the composition of the scaffold’s materials, Fourier transform infrared spectroscopy (FTIR) analysis was performed. The scaffold was ground into power using an agate mortar before compressing into potassium bromide disc. The spectral width ranged from 4000 to 400 cm^-1^ and the spectral resolution of 1 cm^-1^. The spectra were recorded on a FTIR spectrophotometer (Thermo Scientific Nicolet iS5).

### GM release from microspheres and composite scaffolds

The CMs(GM) or CMs(GM)-β-TCP/gelatin composite scaffolds were placed in dialysis bags, and 2 mL of phosphoric acid buffer (pH = 8.0) was added. The dialysis bags were placed in 15 mL of phosphoric acid buffer (pH = 8.0) and oscillated at 37°C. The sampling times of the MSs were 0, 0.5, 1, 1.5, 2, 4, 5, 6, 7, 8, and 24 h. The sampling times of the scaffolds were 0, 1, 2, 3, 4, 5, 6, 7, 24, 48, and 96 h. At each time point, 5 mL of the sample was taken, and 5 mL of fresh phosphate buffer was supplemented. As mentioned above, the GM concentration in the collected samples was determined using derivative UV spectrophotometry. Thus, sustained-release curves of the CMs(GM) and CMs(GM)-β-TCP/gelatin composite scaffolds were obtained.

### Construction of radial segmental bone defect model accompanied by infection

Under aseptic conditions, a longitudinal incision was made on the lateral side of the forelimb of the rabbits after anesthetized with 3% pentobarbital sodium at 20 mg kg^-1^ through ear intravenous injection. The muscle fascia was separated, and the middle and upper parts of the radius were exposed. A 15-mm defect was made using a dental drill, and saline was applied to cool the tissue. A sponge injected with *S*. *aureus* suspension (concentration of 1 × 10^8^ CFU) was placed in the radial defect of the infected group. Then, the incisions were sutured, and the rabbits were kept in individual cages. Four weeks after *S*. *aureus* implantation, the severity of the lesions in the control and infected groups was confirmed using X-rays after anesthesia. Three rabbits from each group were randomly selected and euthanized. Bone and muscle specimens from the bone defect sites were taken and fixed with 4% paraformaldehyde. The specimens were embedded in paraffin and sectioned for hematoxylin and eosin (HE) staining.

### Effects of composite scaffolds on repair of bone defect accompanied by infection *in vivo*

Four weeks after *S*. *aureus* implantation, recanalization of the bone marrow was performed at the defect end, and the composite scaffold was implanted in the experimental group. On the 1st day postoperatively, implantation was confirmed by X-ray examination. Nine weeks after implantation, all the rabbits in the three groups were sacrificed by anesthesia overdose, and the radii were collected for macroscopic observation. Finally, the bone tissues from the bone defect sites were removed and fixed in formalin solution. The specimens were embedded in methyl methacrylate and sectioned for modified van Gieson staining. Briefly, stained in celestine solution blue for 10 min, then rinsed in tap water; stained in hematoxylin solution for 10 min, then rinsed in tap water; acid differentiation for 30s, then rinsed in tap water; stained in working solution for 10 min, then rinsed in tap water; dehydrated in alcohol and cleared in xylol.

### Statistical analyses

All data are reported as the mean ± SD unless otherwise stated. Statistical analyses were performed using the SPSS software, version 16.0 (Chicago, USA).

## Results

### Characterization and bactericidal activity of microspheres

**Fig 2:**
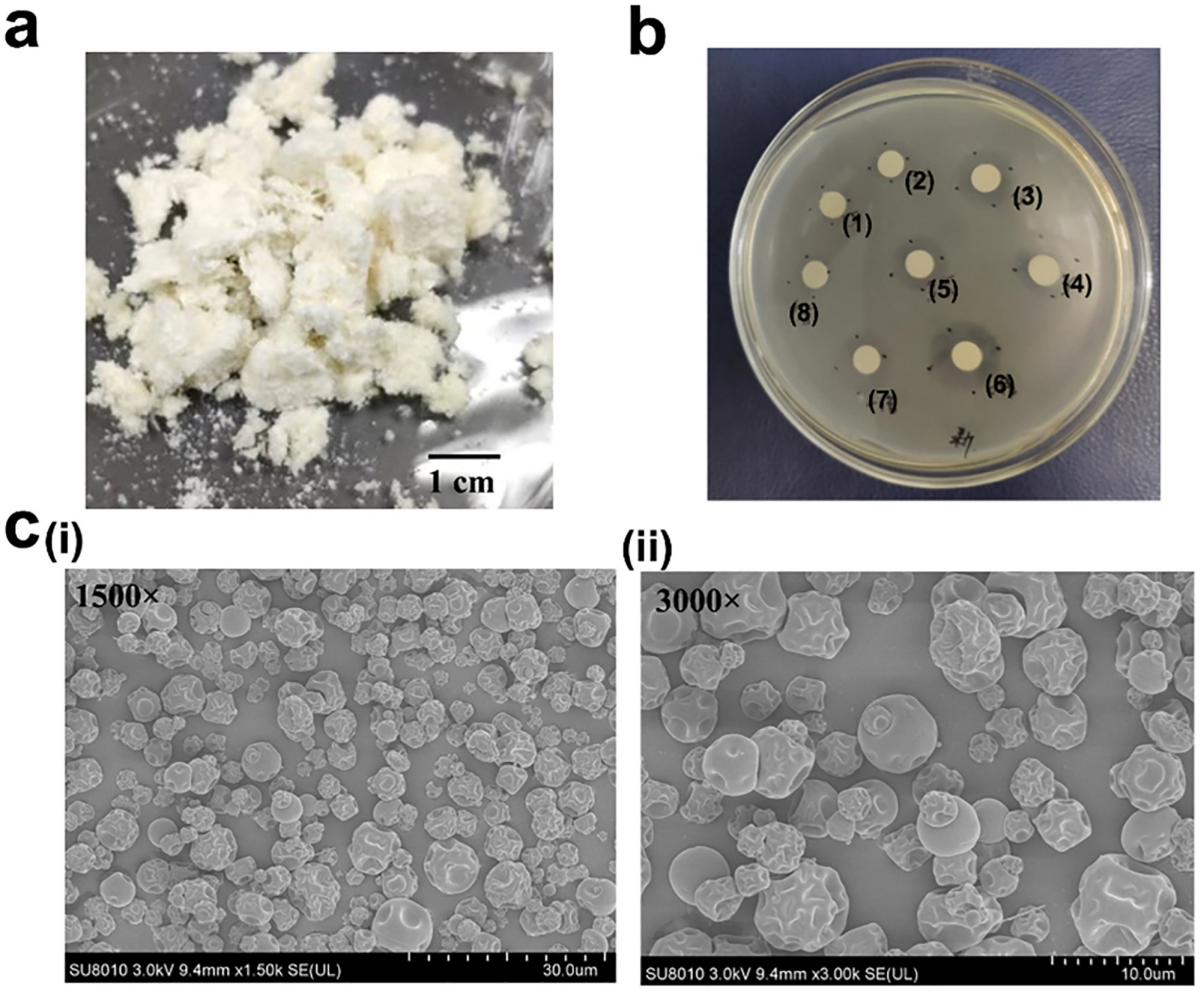
Characterization of microspheres. **a** Macroscopic observation of CMs(GM). **b** The antibacterial activity of the CMs(GM). (1)—(5) represent paper disks contained 15, 20, 25, 30, and 35 μg mL^-1^ GM, respectively. (6)—(8) represent disks contained 1-fold, 2-fold and 4-fold dilutions of CMs(GM) leachate. **c** SEM images of CMs(GM) with (i) scale bar = 30 μm and (ii) scale bar = 10 μm. (CMs: Chitosan microspheres; GM: Gentamycin; β-TCP: β-tricalcium phosphate; SEM: Scanning electron microscopy).

The CMs(GM) were primrose yellow and their mean diameter was 4.51 ± 1.68 μm (see Fig 2a and 2c). A standard curve was constructed to determine the GM concentration. The EE% and DL% of GM in CMs(GM) were 43.76 and 14.53%, respectively. In this work, the antibacterial activity of the CMs(GM) was evaluated through an agar diffusion method. The results revealed that the leachate of CMs(GM) inhibited the growth of *S*. *aureus* in a dose-dependent manner (Fig 2b). The filter paper containing two-fold dilution of leachate presented the similar size of inhibitory zone as the paper containing 30 μg mL^-1^ GM. This indicated that approximately 6 mg GM was released from 100 mg CMs(GM).

### Characterization of composite scaffolds

**Fig 3:**
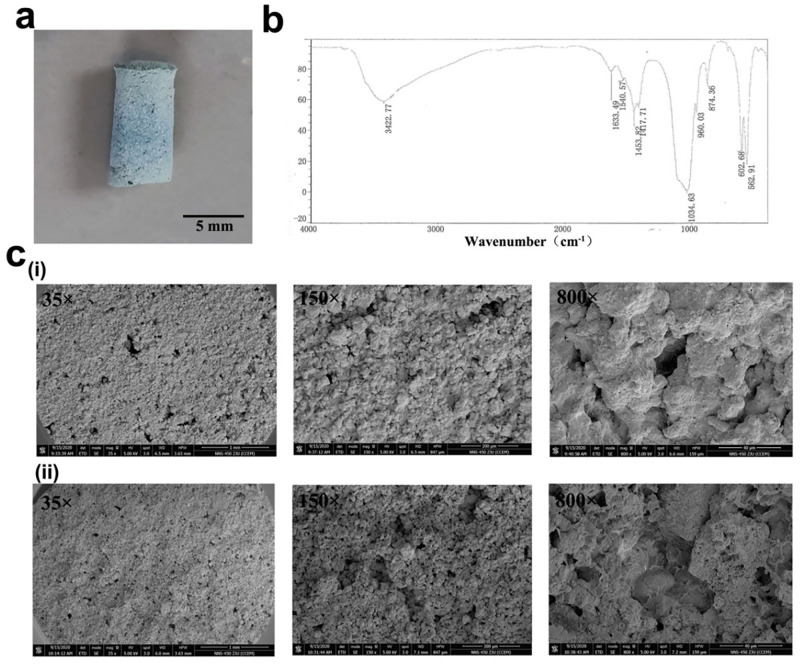
Characterization of CMs(GM)-β-TCP/gelatin composite scaffolds. **a** Macroscopic observation of CMs(GM)-β-TCP/gelatin composite scaffolds. **b** FTIR spectra for the β-TCP/gelatin composite scaffolds. **c** SEM images of (i) CMs-β-TCP/gelatin composite scaffolds (left to right: Scale bars = 1 mm, 200 μm, and 50 μm, respectively) and (ii) CMs(GM)-β-TCP/gelatin composite scaffolds (left to right: Scale bars = 1 mm, 200 μm, and 50 μm, respectively). (CMs: Chitosan microspheres; GM: Gentamycin; β-TCP: β-tricalcium phosphate; SEM: Scanning electron microscopy; FTIR: Fourier transform infrared spectroscopy).

The CMs(GM)-β-TCP/gelatin composite scaffolds were blue (see Fig 3a). The FTIR spectra of β-TCP/gelatin scaffold are presented in Fig 3b. Beta-TCP reveals peaks at 562.91 cm^-1^, 602.68 cm^-1^, 960.03 cm^-1^ and 1034.63 cm^-1^ (be belong to PO_4_^3-^). Gelatin reveals peak at 1540.57 cm-^1^ (be belong to N-H (II) bond). Fig 3c show the morphologies of the CMs(GM)-β-TCP/gelatin composite scaffolds. The inner structure of the scaffold was arranged in the shape of interconnected pores, and the apertures were uniform in size, which may provide adequate space for cell adhesion and proliferation and facilitate the transport of nutrients and metabolic waste. The compressive strength of the scaffold was 0.82 ± 0.05 MPa, and the load at the time of the fracture was 10.27 ± 0.65 N.

### *In vitro* release of GM from microspheres and composite scaffolds

**Fig 4:**
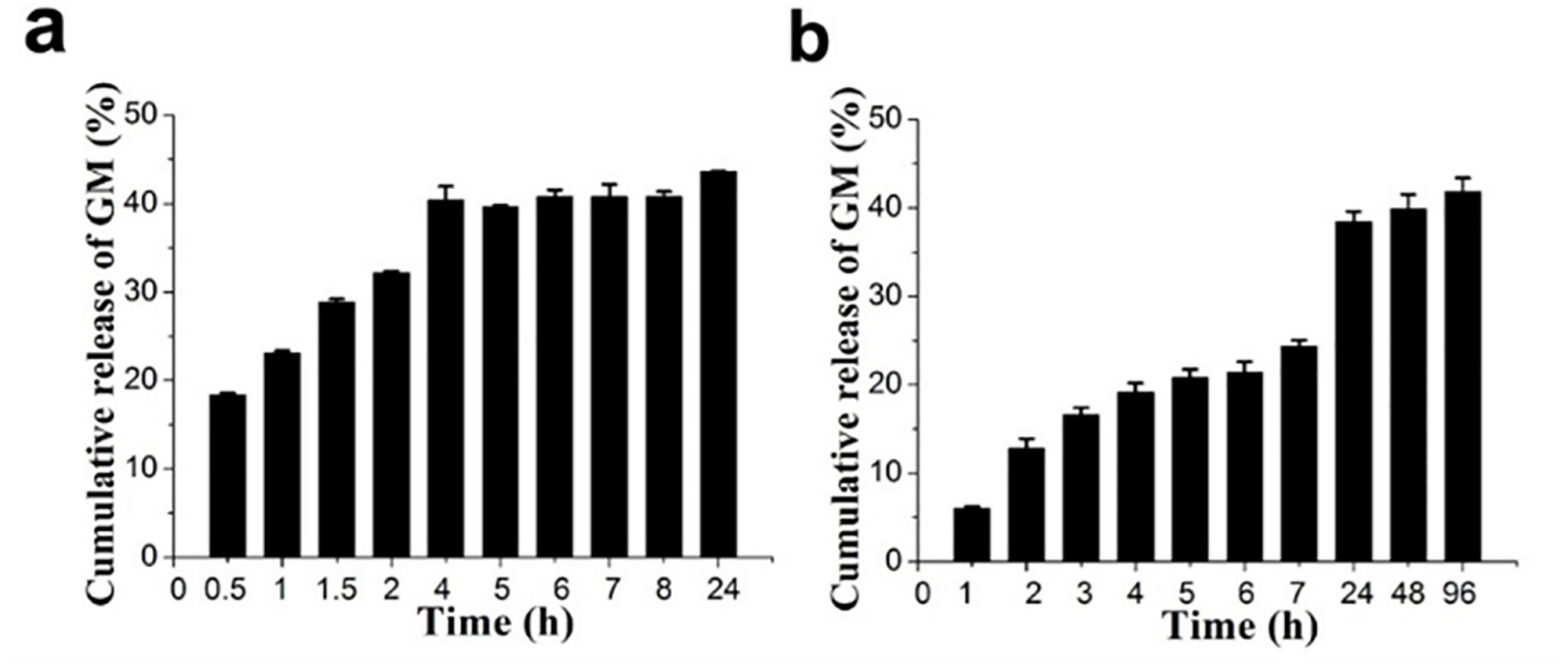
Cumulative release curves of (a) CMs(GM) and (b) CMs(GM)-β-TCP/gelatin composite scaffolds. (CMs: Chitosan microspheres; GM: Gentamycin; β-TCP: β-tricalcium phosphate).

The release of GM from CMs(GM) and CMs(GM)-β-TCP/gelatin composite scaffolds was monitored for 24 h and 96 h, respectively. The cumulative release curves (Fig 4) show that both the MSs and composite scaffolds reached a sustained slow-release phase after the initial rapid release. The release curve of CMs(GM) reached a plateau after 4 h, and the cumulative release reached 43.61% during this period. The release curve of CMs(GM)-β-TCP/gelatin composite scaffolds reached a plateau after 24 h, and the cumulative release reached 41.86% during this period. The results showed that CMs(GM)-β-TCP/gelatin composite scaffolds could rapidly release GM to inhibit bacterial proliferation in the initial stage of implantation and sustainably release GM in the later stage to provide a suitable internal environment for defect repair.

### Construction of radial segmental bone defect model accompanied by infection

**Fig 5:**
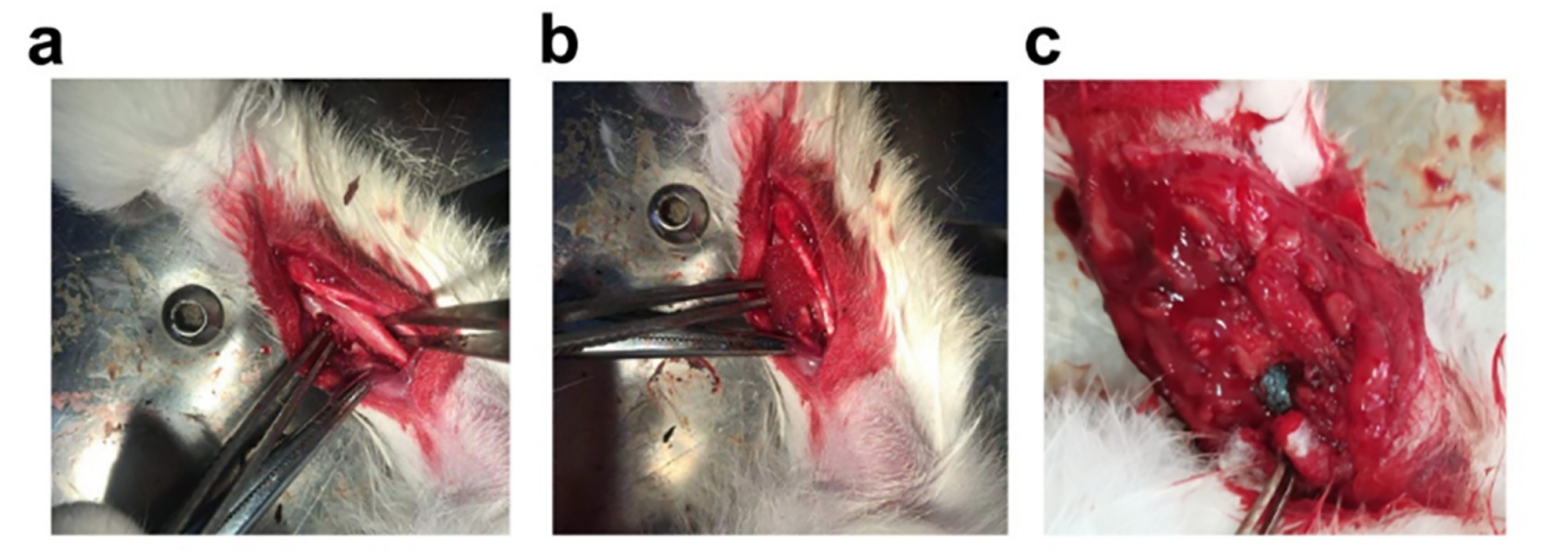
Construction of (a) radial segmental bone defect models (control group; n = 6 rabbits) and (b) rabbit radial segmental bone defect models accompanied by infection (infected group; n = 6 rabbits); (c) recanalization of bone marrow and implantation of CMs(GM)-β-TCP/gelatin composite scaffolds in the 4th week after model generation (experimental group; n = 3 rabbits). (CMs: Chitosan microspheres; GM: Gentamycin; β-TCP: β-tricalcium phosphate).

**Fig 6:**
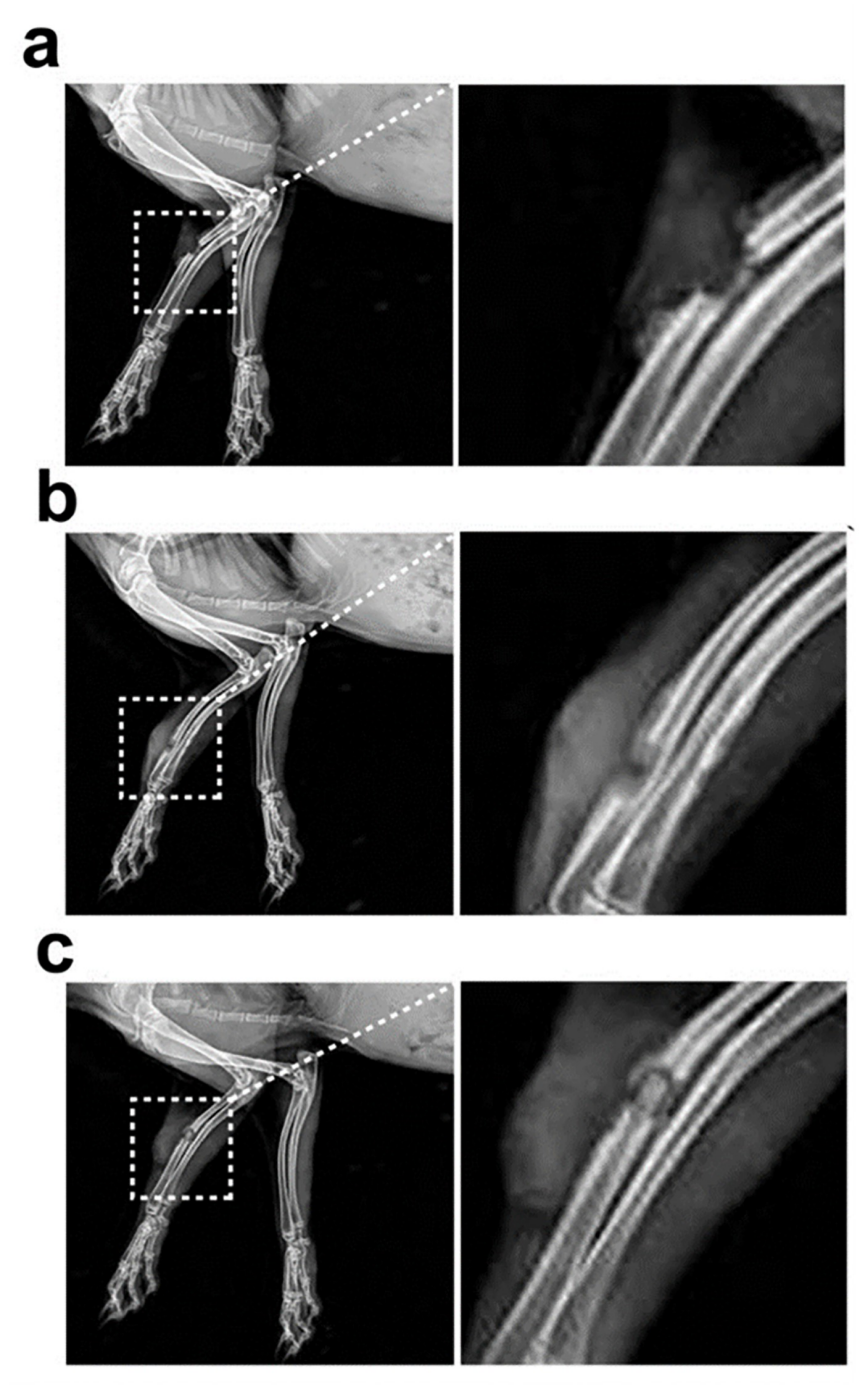
Radiograph results of (a) control group, (b) infected group at 4th week after modeling, and (c) experimental group on the 1st day after implantation (n = 3 rabbits per group).

**Fig 7:**
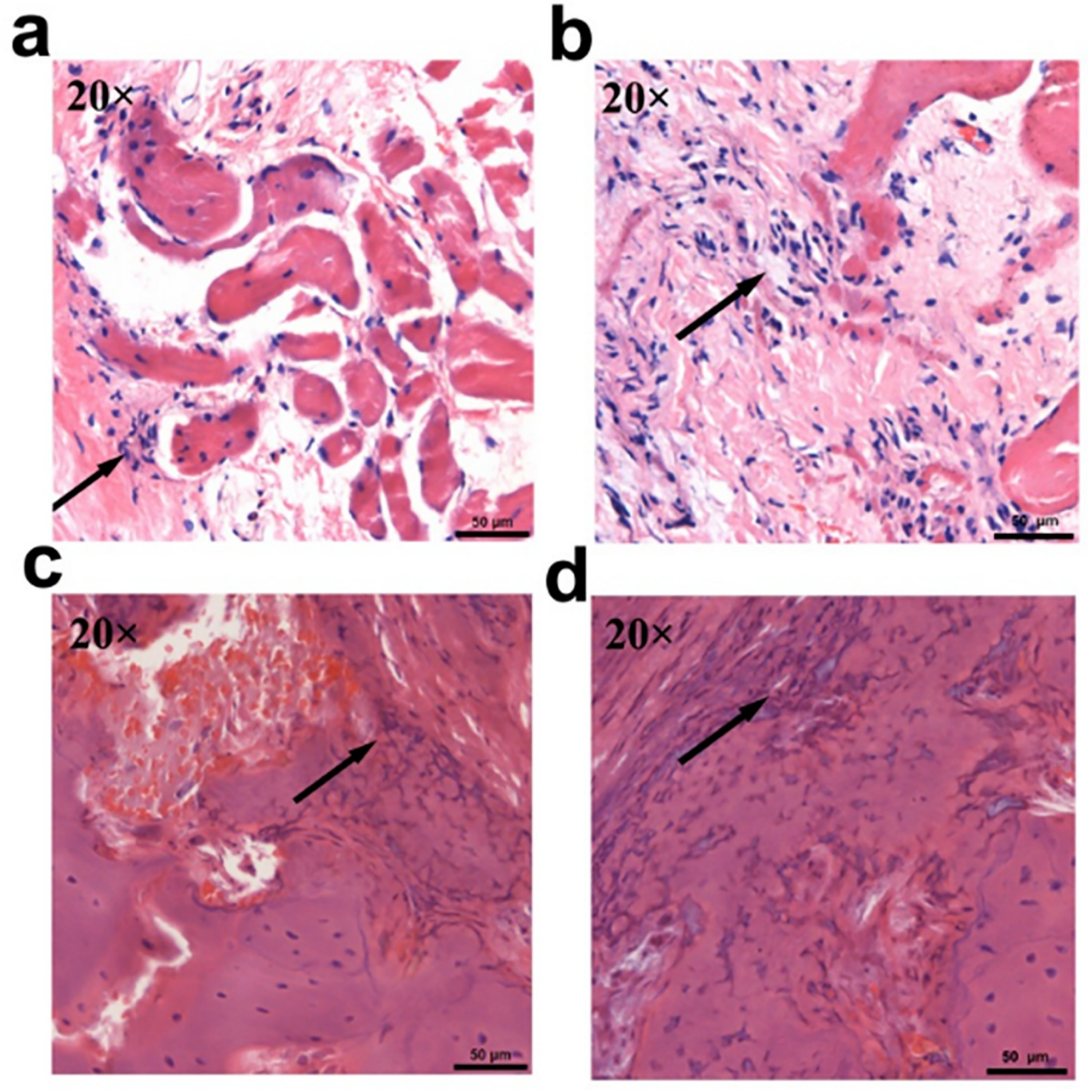
Histological observations of (a) muscle and (c) bone tissue in the control group and (b) muscle and (d) bone tissue in the infected group in the 4th week after model generation. The black arrows represent inflammatory cells (scale bar = 50 μm; n = 3 rabbits per group). (CMs: Chitosan microspheres; GM: Gentamycin; β-TCP: β-tricalcium phosphate).

As described in the Materials and Methods section, rabbit radial segmental bone defect model and radial segmental bone defect model accompanied by infection were successfully established (Fig 5a and 5b). On the 1st day postoperatively, compared with the control group bone defects alone, the wounds of rabbits inoculated with *S*. *aureus* were more swollen. In the 4th week postoperatively, the X-ray results showed that the defect ends of the radius in the control and infected groups were both smooth, and no new tissue was formed. Tissue swelling, inflammatory reactions, periosteal hyperplasia, and destruction were observed around the defect (Fig 6a and 6b). HE staining results showed that bone trabeculae disappeared in the control and infected groups. Muscle fiber necrosis, striation disappearance, nuclear inward migration, and interstitial widening were observed in the skeletal muscle. Inflammatory cells infiltrated some bone and skeletal muscle tissues, with red blood cells scattered between them (Fig 7).

### Effects of composite scaffolds on repair of bone defect accompanied by infection *in vivo*

**Fig 8:**
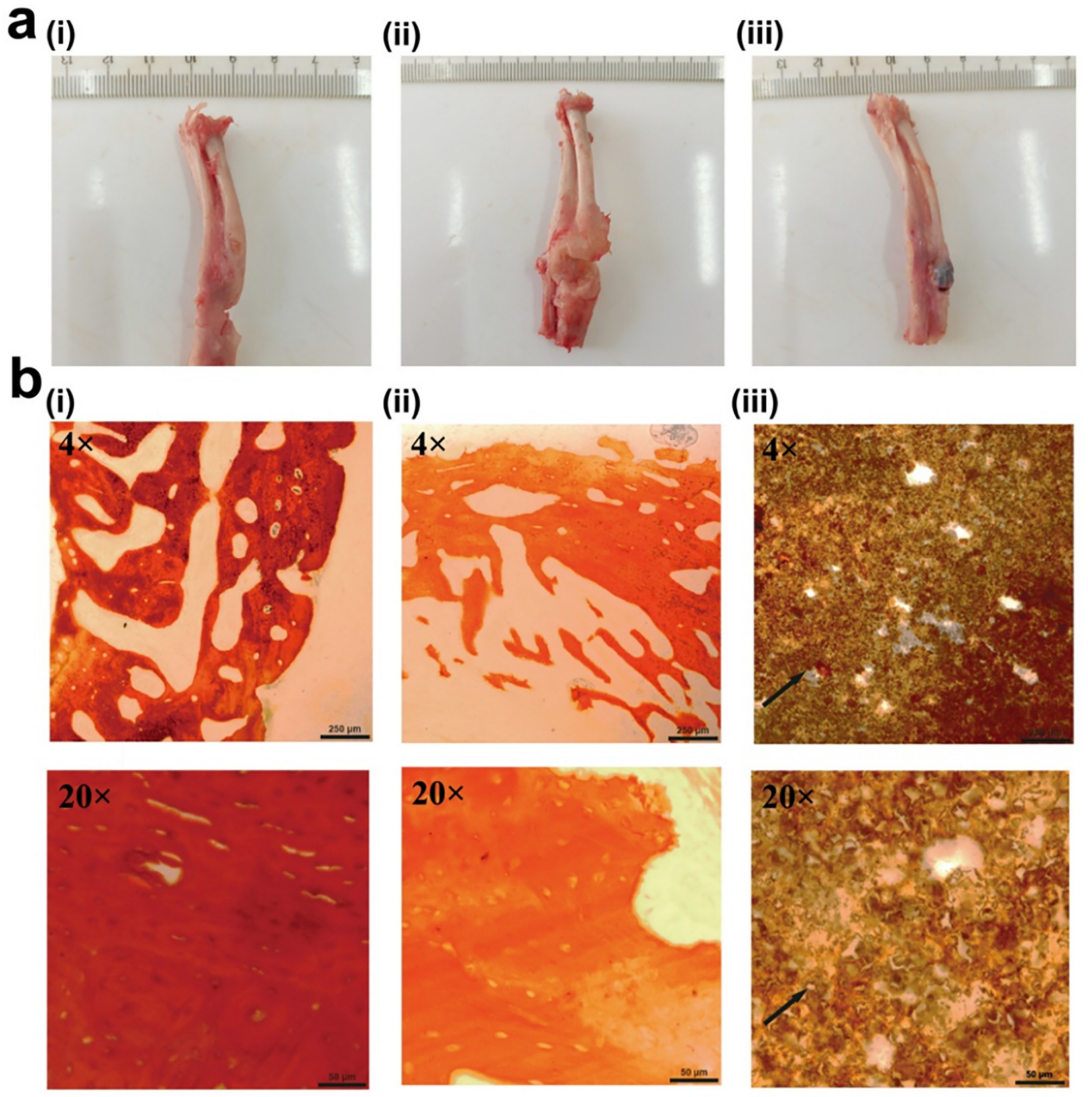
Effects of composite scaffolds on repair of bone defect accompanied by infection *in vivo*. **a** Macroscopic observations of radii in the (i) control, (ii) infected, and (iii) experimental groups in the 13th week after model generation (n = 3 rabbits per group). **b** van Gieson staining of bone tissues in the (i) control, (ii) infected, and (iii) experimental groups in the 13th week after model generation. The black arrows represent newly formed bone (top to bottom: Scale bars = 50 μm and 250 μm, respectively; n = 3 rabbits per group). (CMs: Chitosan microspheres; GM: Gentamycin; β-TCP: β-tricalcium phosphate).

Four weeks after *S*. *aureus* implantation, recanalization of the bone marrow was performed at the defect end, and the composite scaffold was implanted in the experimental group (Fig 5c). On the 1st day postoperatively, X-ray examination confirmed successful implantation (Fig 6c). The rabbits in the three groups were fed for another nine weeks and then sacrificed. The radii were excised for general observations. The composite scaffolds in the experimental group were fused with the bone tissues, whereas no new tissue was formed in the defect areas of the control and infected groups (Fig 8a). Furthermore, van Gieson staining showed new bone formation in the composite scaffolds (Fig 8b).

## Discussion

The problem of tissue infection caused by microbial contamination, such as bacteria, poses a challenge to the treatment and recovery of bone defects. Bacterial osteomyelitis (BO) is a common infection of bone tissue. The affected areas have poor self-healing ability due to the decreased blood flow caused by chronic infection. The conventional treatments of BO include extensive debridement of all necrotic and systemic administration of antibiotics for weeks [28]. The antibiotics treatment is notoriously difficult, in part because of the development of widespread antibiotics resistance [29]. Moreover, BO triggers pathological bone remodeling, which in turn lead to sequestration of infectious foci [29]. The etiologic agents of BO, such as *S*. *aureus*, may colonize the surface of the objects and form biofilms [30]. The limited blood supply and bacteria film prevent the accumulation of antibiotics in the infected area [10].

Currently, the developments in tissue engineering have offered alternatives to traditional therapies. The common strategy includes local controlled release of antibiotics. As tailorable platforms, MSs are often used as drug carriers in the treatment of bone defects [31,32]. MSs provide physicochemical gradients via spatiotemporal release of antibiotics. MSs fabricated using CS can also play an antibacterial role in the degradation process. In this study, we encapsulated GM in CS to obtain CMs(GM). The results of the antibacterial activity assay showed that the leachate of CMs(GM) inhibited the growth of *S*. *aureus* in a dose-dependent manner. The release curve of CMs(GM) reached a sustained slow-release phase after 4 h, which can provide the requisite antibiotic delivery and control infection.

However, particles of small sizes may be easily cleared by capillaries and lymphatic vessels, which affect the function of MSs. Additionally, the ideal biomaterials should enhance bone regeneration when the infection is controlled. And the combination of MSs and scaffolds is an effective preparation scheme [33–35]. PMMA has traditionally been utilized as filler to obliterate dead space from bone loss, although it does not provide environment for bone to regrow [21]. Bone is a complex tissue mainly composed of calcium phosphate and collagen. Scaffolds for osteogenesis should mimic bone composition and provide the requisite mechanical strength. β-TCP is a biomaterial with good osteoconductive and osteoinductive properties that can be degraded *in vivo* [36]. As a natural protein, gelatin can promote cell adhesion and proliferation and can help improve the properties of inorganic materials [37,38]. Prior work by Huang et al demonstrated gelatin promote the adhesion and growth of cells [38]. In this study, the GM-loaded CSs were incorporated into β-TCP/gelatin composite scaffolds to achieve local delivery of GM at the defect site and further stabilize the initial drug release rate. The composite scaffold presented the compression strength of 0.82 ± 0.05 MPa, which was lower than the mechanical strength value of trabecular bone (2–20 MPa). Nevertheless, scaffolds with similar or lower compression strength reported in literature were able to provide adequate support as templates during the first phase of bone generation [39,40]. The composite scaffolds were biodegraded and replaced by the new bone. Hence, the combination of β-TCP and gelatin displays biological and functional characteristics that are similar to those of bone.

On the other hand, pores are important for tissue regeneration, which is necessary for cell migration and proliferation. The interconnected pores of the composite scaffold were uniform in size, which favored direct osteogenesis. We also investigated the therapeutic efficacy in rabbits of BO. New tissues were formed in the defected areas where the composite scaffolds were fused with the bone. All these results proved that CMs(GM)–β-TCP/gelatin composite scaffolds had contribution to the efficacious management of BO after surgical debridement. The composite scaffolds exhibited good osteoconduction and were progressively replaced by new formed bone.

The results obtained in this study are applicable only for rabbit models; they should be further verified in larger animal models with physiological and immune similarities to humans. This could provide evidence for safety and efficiency.

## Conclusion

CMs(GM)–β-TCP/gelatin composite scaffolds were successfully fabricated. *In vitro* assessments showed that the composite scaffolds could release GM rapidly in the initial stage to inhibit bacterial proliferation and continued to release GM in the late stage to maintain a suitable environment. The interconnected pores in the scaffold could facilitate the transport of nutrients and metabolic waste. *In vivo* experiments demonstrated that CMs(GM)–β-TCP/gelatin composite scaffolds promoted the formation of new bone.

## Supporting information

S1 File The compressive strength of CMs(GM)-β-TCP/gelatin composite scaffolds. (XLSX)

S2 File The cumulative release curves of CMs(GM). (OPJ)

S3 File The cumulative release curves of CMs(GM)-β-TCP/gelatin composite scaffolds. (OPJ)
